# Should the tip-apex distance (TAD) rule be modified for the proximal femoral nail antirotation (PFNA)? A retrospective study

**DOI:** 10.1186/1749-799X-8-35

**Published:** 2013-10-17

**Authors:** Andrej N Nikoloski, Anthony L Osbrough, Piers J Yates

**Affiliations:** 1 Fremantle Hospital Orthopaedic Unit, Fremantle Hospital, Level 6, B Block, Alma Street, Fremantle, Western Australia 6160, Australia; 2 Orthopaedic Surgery, Fremantle Hospital, University of Western Australia, Crawley, Western Australia 6009, Australia; 3 Fremantle and Kaleeya University Hospitals, East Fremantle, Western Australia 6158, Australia

**Keywords:** Proximal, Femur, Fracture, Intramedullary, Nail, Fixation, PFNA, TAD

## Abstract

**Background:**

Unstable proximal femoral fractures are common and challenging for the orthopaedic surgeon. Often, these are treated with intramedullary nails. The most common mode of failure of any device to treat these fractures is cut-out. The Synthes proximal femoral nail antirotation (PFNA) is unique because it is the only proximal femoral intramedullary nail which employs a helical blade in lieu of a lag screw. The optimal tip-apex distance is 25 mm or less for a dynamic hip screw. The optimal blade tip placement is not known for the PFNA.

**Aim:**

The aim of this study is to determine if the traditional tip-apex distance rule (<25 mm) applies to the PFNA.

**Method:**

A retrospective study of all proximal femoral fractures treated with the PFNA in Western Australian public teaching hospitals between August 2006 and October 2007 was performed. Cases were identified from company and theatre implant use records. Patient demographic data was obtained from hospital records. Fractures were classified according to Arbeitsgemeinschaft für Osteosynthesefragen/Association for the Study of Internal Fixation. Fracture reduction, distal locking type and blade position within the head (tip-apex distance and Cleveland zone) were recorded from the intraoperative and immediate postoperative radiographs. Postoperative radiographs obtained in the routine treatment of patients were studied for review looking primarily for cut-out. Clinical outcomes were measured with the Oxford hip score.

**Results:**

One hundred eighty-eight PFNAs were implanted during the study period, with 178 cases included in this study. Ninety-seven patients could be followed up clinically. There were 18 surgical implant-related failures (19%). The single most common mode of failure was cut-out in six cases (6.2%). Three cut-outs (two medial perforation and one varus collapse) occurred with tip-apex distance (TAD) less than 20 mm. There was no cut-out in cases where the TAD was from 20–30 mm. There were three implant-related failures (nail fracture, missed nail and loose locking screw), four implant-related femoral fractures, two non-unions, two delayed unions and one loss of reduction.

**Conclusion:**

The PFNA is a suitable fixation device for the treatment of unstable proximal femoral fractures. There were still a relatively large number of cut-outs, and the tip-apex distance in the failures showed a bimodal distribution, not like previously demonstrated with dynamic hip screw. We propose that the helical blade behaves differently to a screw, and placement too close to the subchondral bone may lead to penetration through the head.

## Introduction

Unstable proximal femoral fractures are common and challenging for the orthopaedic surgeon. The aim of the surgical treatment of these fractures is to achieve stable fracture fixation that will allow early weight bearing. Many different devices have been developed, yet mechanical failures still occur. Cephalomedullary nails are now favoured in most Western Australian teaching hospitals for the treatment of unstable proximal femur fractures. The complication rate is quoted as being from 15% to 20%, with the most common mode of failure being screw or blade cut-out [1-3]. In biomechanical studies, the spiral blade of the Synthes proximal femoral nail antirotation (PFNA; Synthes GmbH, Oberdorf, Switzerland) has shown a superior cut-out resistance, which may translate into fewer cut-outs in the clinical setting [4,5]. The technique guide for this implant suggests a distance from the blade tip to the joint level of 10 mm in the anteroposterior and lateral projections [6]. This corresponds to a tip-apex distance of 20 mm. When using a sliding hip screw and plate construct, a tip-apex distance (TAD) of less than 25 mm and centre-centre positioning has been established as a major factor to minimise the risk of cut-out [7]. There is little similar data on the optimal placement of the blade for intramedullary devices and none for blade based intramedullary devices such as the PFNA.

### Aim

The aim of the study is to determine if the traditional tip-apex distance rule (<25 mm) applies to a helical blade device.

### Patients and methods

We retrospectively identified 187 patients who had 188 fractures treated with the Synthes PFNA at the three Western Australian tertiary teaching hospitals between August 2006 and October 2007. These were identified via company and hospital theatre records. Ten patients were excluded because they either had the nail implanted as fracture prophylaxis for a tumour or as an intracapsular or diaphyseal region fracture.

The medical records and picture archiving system (AGFA Impax 5, Ridgefield Park, NJ, USA) of the remaining 178 patients were accessed to obtain clinical data and radiographs for analysis. Preoperative radiographs were used to classify fractures according to Arbeitsgemeinschaft für Osteosynthesefragen/Association for the Study of Internal Fixation (AO/ASIF). Immediate postoperative radiographs, when available, were analysed for reduction quality and blade tip position as per Cleveland and TAD [7,8]. All measurements were performed by the chief author. In those cases where an immediate (day 1) postoperative X-ray was not available, this data was obtained from the fluoroscopy images. Operation notes were reviewed to confirm that the surgery followed the manufacturer's surgical technique manual [6]. All subsequent X-ray images for each patient were reviewed looking primarily for cut-out (varus collapse or medial perforation) as well as any other radiologically apparent complication. The clinical follow-up consisted of outpatient clinical follow-up with Oxford hip score for those patients that could attend. In our hospitals, we use the Oxford hip score routinely to assess pain and function in patients with hip pathology.

Data was analysed for descriptive statistics using SPSS statistical software version 11.0 (SPSS Inc. Chicago, IL, USA). We used Pearson's chi-squared test or Fisher's exact test for comparing differences in categorical variables.

## Results

**Figure 1:**
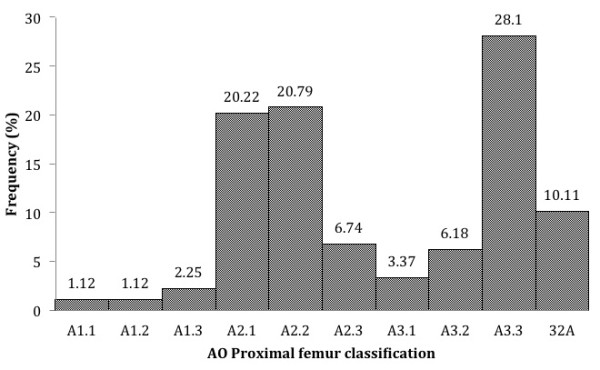
AO/ASIF classification.

Patient demographics and characteristics are provided in Table 1. We analysed the preoperative radiographs to classify fractures according to AO/ASIF (Figure 1). The majority, 152 (95.5%), were unstable fracture types; of these, 31A2 (47.7%) and 31A3 (37.6%) were dominant. There were 13 (7.3%) fractures of the subtrochanteric region (31A3.3, 32A).

**Table 1:** Patient characteristics

	**Value**
Age (years)	
Mean	81.5
Range	36 to 99
Gender (*n* (%))	
Female	128 (71.9)
Male	50 (28.1)
Side (*n* (%))	
Left	107 (60.1)
Right	81 (45.5)

The nail used was short (240 mm) in 136 (76.4%) and long (>240 mm) in 42 (23.6%) cases. Distal locking was static in 146 (82%) and dynamic in 32 (18%) cases. Postoperative treatment for all patients was full weight bearing.

Postoperative radiographs were analysed for reduction quality, TAD and Cleveland zone. Reduction quality was graded as anatomical, near-anatomical and non-anatomical and was assessed by the chief investigator. The reduction was anatomical in 123 (69.1%), near-anatomical in 45 (25.2%) and non-anatomical in 10 (5.6%) cases.

**Figure 2:**
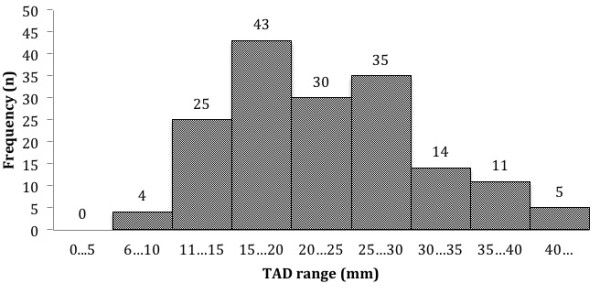
TAD distribution.

The TAD was assessed on the immediate postoperative radiographs, using the method described by Baumgaertner [9]. The TAD ranged from 7 to 45 mm, with 96 (53.9%) cases under 25 mm (Figure 2).

**Figure 3:**
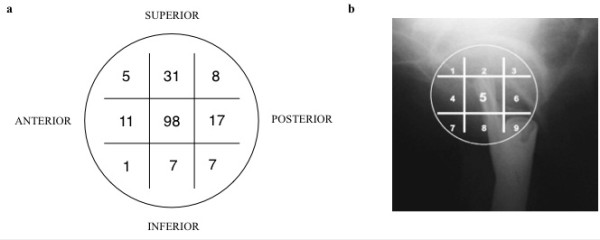
Cleveland index distribution (a) and Cleveland zones (b).

The location of the blade within the head was recorded as per the Cleveland method, on a lateral X-ray of the femoral head, divided into nine sections [8]. The Cleveland zone 5 (centre-centre) was the most common placement of the tip of the blade on postoperative radiographs, accounting for 98 (55.1%) of the cases. The second most common location was zone 2 (centre-superior) with 31 (17.4%) cases (Figure 3).

The average time to clinical follow-up was 10 months. Within the follow-up period, 45 (24%) patients died of medical comorbidities, and 36 (19%) patients could not be contacted or were unable or unwilling to attend the outpatient clinic. The total number of patients who could be clinically followed-up was thus 97. For the patients who had clinical follow-up, the mean Oxford hip score was 27, ranging from 12 to 45 (out of 48).

### Complications

**Figure 4:**
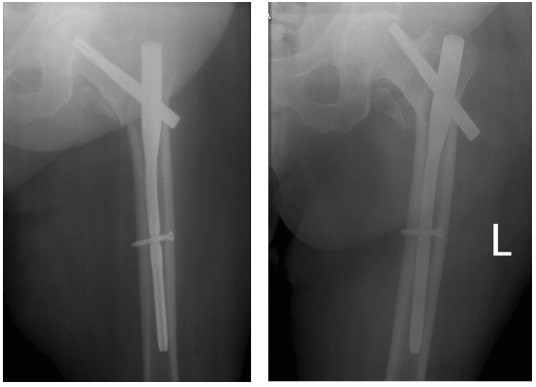
**Cut-out with TAD 42 mm.** Blade has migrated superiorly.

**Figure 5:**
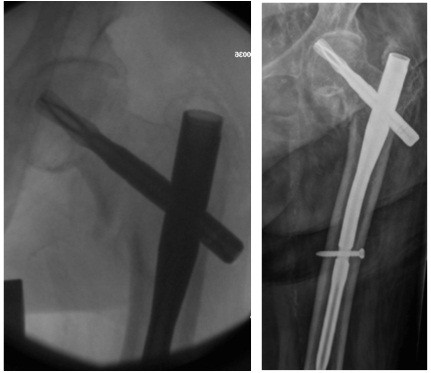
**Fluoroscopy image of PFNA with TAD of 9 mm.** Blade has migrated axially.

**Figure 6:**
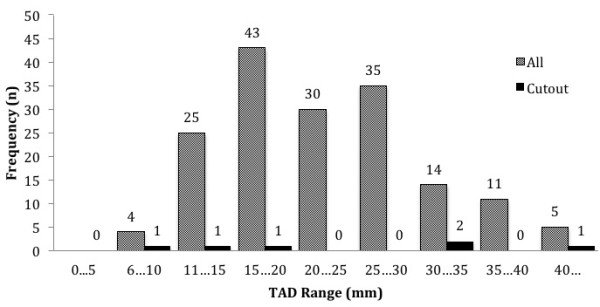
Distribution of TAD in cut-out and all cases.

There were 18 out of 97 (18.6%) surgical implant-related complications identified in the study. There were six (6.2%) cases of cut-out. We saw two patterns of cut-out: cephalad cut-out (varus head collapse) and axial cut-out (medial or anterosuperior migration). Cephalad cut-out occurred in 4 (4.1%) and axial cut-out (medial perforation) in 2 (2.1%) of 97 cases (Figures 4 and 5). All were in unstable fracture types, four in fracture type 31A2 and two in 31A3. The reduction was anatomical or near anatomical in five of the cases and non-anatomical in one case. Three of the cephalad cut-outs had a TAD greater than 30 mm, and in one case, it was 15 mm. The two axial cut-outs (medial perforations) occurred where the TAD was less than 20 mm. No failures occurred where the TAD was in the interval of 20 to 30 mm (Figure 6).

When analysed in three groups: TAD 0–20 mm, TAD 20–30 mm, TAD >30 mm, there was a statistically significant difference in the frequency of cut-out between the cases with TAD 0–20 mm and TAD 20–30 mm (*p* = 0.0293), but not between TAD 0–20 mm and TAD >30 mm (*p* = 0.3707) (Table 2).

**Table 2:** Tip-apex distance and cut-out frequency

**TAD (mm)**	**Cut-out ( *n *)**	**No cut-out ( *n *)**
0–20	3	31
20–30	0	74
>30	3	73

The Cleveland zone distribution of the blades which cut-out were zone 5 (centre, centre) in three cases, zone 2 (centre, superior) in one case, zone 4 (centre, anterior) in one case and zone 1 (superior, posterior) in one case. In the cases of failure associated with a TAD 0–20 mm, the position of the blade was in zone 5 (centre, centre). There was no statistically significant difference in cut-out rates between the nine different zones (*p* = 0.565).

Other complications in the order of frequency were three intraoperative femur fractures (1.7% of all cases), two periprosthetic fractures (distal to the tip of the nail), two cases of delayed union (1.1%) and two cases of non-union (1.1%). The intraoperative femur fractures all occurred when using long nails. Both non-unions occurred in subtrochanteric fractures. There were no infections.

## Discussion

Optimal fixation of proximal femoral fractures is still controversial. There is no evidence in the literature demonstrating that an intramedullary nail is superior to extramedullary devices, such as a DHS, when used for stable fracture types [1,9-12]. Some studies comparing the gamma nail to the DHS show an increased rate of complications including femur fracture with the use of an intramedullary device [13]. Biomechanically, intramedullary devices have been shown to be superior for unstable fracture types. A recent prospective randomised study comparing the PFNA to the DHS in mainly unstable fracture patterns found no statistically significant difference in complications [13]. The predominant trend in Western Australian tertiary centres is to use the PFNA in unstable fractures of AO/ASIF type 31A2 and 31A3. We found that our complication rate is comparable to other studies of the PFNA [5,14-18], but that it is also similar to that with older nail designs [3]. The change to a helical blade with the introduction of the PFNA (and TFN in other markets) was intended to reduce the likelihood of cut-out and to eliminate the occurrence of the Z-effect mode of failure of the old PFN. Several biomechanical studies have supported this [4,19]. The phenomenon of cut-out has however not been eliminated and is in fact still the most common mode of failure.

We had 6 (6.2%) cases of cut-out in our series of 178. Several clinical studies report cases of cut-out with the PFNA. Brunner et al. reported 3 (25%) cases of cut-out out of 12 [18]. They raised the possibility that the mode of cut-out of the PFNA may be related to its helical blade design, which may result in medial perforation of the subchondral bone. Mereddy et al. had 2 (3.2%) cases in their series of 62 [17]. They reported their TAD as <20 mm in 79% of the total cases but did not report what the TAD was in cases of cut-out. Penzkofer et al. reported 3 (4.5%) cut-outs in their cohort of 66 pertrochanteric and subtrochanteric fractures treated with PFNA [20]. A randomised trial by Wild et al. compared the PFNA with a two-screw type intramedullary nail (Targon PF, Aesculap, Tuttlingen, Germany) and had 3 (7.5%) cases of cut-out in the PFNA group of 40 and 2 (5%) in their group of 40 treated with the Targon PF nail [21]. Takigami et al. had 1 (2%) cut-out in their series of 50 pertrochanteric fractures treated with PFNA for unstable fractures [22].

Several authors however have reported much lower rates of cut-out, such as Liu et al. and Pu et al., who had no cases of cut-out in their series of 125 and 87, respectively [23,24]. Liu et al. attributed their lack of cut-out to their more conservative post-op rehabilitation regime. Pu et al. reported an average TAD of 16.8 mm, but suggested not putting the helical blade tip closer than 10 mm from subchondral bone and using a shorter blade in order to avoid head perforation. Simmermacher have the largest series to date, with a reported cut-out rate of 4 (1.2%) out of 313 cases [5].

### Optimal blade position

It has been assumed that following the rules established for DHS placement should yield similarly good results with the PFNA. The technique guide for the Synthes PFNA suggests inserting the guide wire to 5–10 mm from subchondral bone on the AP and lateral views, which would yield a TAD of 10–20 mm in the case of centre-centre positioning. Our study is one of several recently that show that cut-out still occurs, despite a tip-apex distance that would be considered ideal for a sliding hip screw. In our series, three cut-outs (two medial perforations and one cephalad cut-out) occurred in cases where the TAD was less than 25 mm and with centre-centre positioning of the tip of the blade. There were no cut-outs in the range of 20–30 mm, and this would be considered ‘too far’ from the apex when using a sliding hip screw such as a DHS. The other three cut-outs were seen in cases where the TAD was more than 30 mm. The failures in these three cases were cephalad cut-out.

One hypothesis is that due to the different geometry of the blade compared to a threaded tip screw, the blade ‘behaves’ differently under load; this potentially results in medial perforation or axial cut-out when inserted too close to the sub-chondral bone. In Simmermacher's large multicentre series, the phenomenon of medial blade migration was attributed to patients falling directly onto the trochanteric region, presumably axially loading the head component of the implant [5]. They did not report their target TAD or the TAD in those cases of cut-out. A recent biomechanical study by Born et al. comparing threaded screw and helical blade constructs in a model of pertrochanteric fracture fixation using polyurethane femoral heads found that the blade device is more prone to cut-out [25]. This is in contrast to previous biomechanical studies [4,19]. The Born et al. testing set-up is unique in that it multiaxially loads the constructs, alluding to the likely reason for the clinical observations of axial migration of helical blades within the head. This study proposes that the blade device, due to its shape, presents a lesser contact surface to the bone in the axial direction. They report an axial contact surface of 75 mm^2^ for the PFNA blade and 300 mm^2^ for the gamma 3 screw. In a recent trial comparing the PFNA with the gamma 3 nail involving 136 unstable proximal femoral fractures, Xu et al. found no cut-out in both groups and similar overall outcomes [26].

We want to raise awareness of the possibility that the helical blade behaves differently to a screw in the femoral head and that following the traditional tip-apex distance recommendation needs to be further validated and even modified when using a blade, due to the risk of medial perforation (axial cut-out). Our results suggest that the tip-apex distance for helical blade-based proximal femoral nails should be 20–30 mm. Zhou and Chang wrote a letter to the editor coming to a similar conclusion based on their experiences from their centre and analysis of the literature [27]. In their opinion, the optimal tip-apex distance for the helical blade should be 20–25 mm.

## Conclusion

We believe that the TAD rule of <25 mm should not apply for the PFNA. We suggest avoiding a TAD <20 mm due to possible axial cut-out (medial migration) and avoiding a TAD >30 mm to avoid cephalad cut-out. We would recommend that the surgical technique guide for the Synthes PFNA be revised to take this into account.

We acknowledge that the study is limited by its retrospective design, short period of follow-up, as well as a large number of patients lost to follow-up. Some of these downfalls are inherent in the population as elderly patients with hip fractures have a higher incidence of comorbidities and thus medical complications subsequent to their fall and surgery.

## Abbreviations

PFNA: Proximal femoral nail antirotation; TAD: Tip-apex distance.

## Competing interests

The senior author (PJY) is a member of the AO Foundation faculty and has received research funding and department grants from Synthes. This study was not financed or supported by any company or organization. The other authors have no competing interests.

## Authors' contributions

ANN designed the study, collected and analysed the data and drafted the manuscript. ALO collected the data. PJY conceived the study and assisted in drafting the manuscript. All authors read and approved the final manuscript.
